# Stable Isotope Signatures of Middle Palaeozoic Ahermatypic Rugose Corals – Deciphering Secondary Alteration, Vital Fractionation Effects, and Palaeoecological Implications

**DOI:** 10.1371/journal.pone.0136289

**Published:** 2015-09-03

**Authors:** Michal Jakubowicz, Blazej Berkowski, Matthias López Correa, Emilia Jarochowska, Michael Joachimski, Zdzislaw Belka

**Affiliations:** 1 Institute of Geoecology and Geoinformation, Adam Mickiewicz University, Poznań, Poland; 2 Institute of Geology, Adam Mickiewicz University, Poznań, Poland; 3 GeoZentrum Nordbayern, Universität Erlangen-Nürnberg, Erlangen, Germany; 4 German University of Technology in Oman (GUtech), Halban Campus, Muscat, Sultanate of Oman; 5 Isotope Laboratory, Adam Mickiewicz University, Poznań, Poland; Union College, UNITED STATES

## Abstract

This study investigates stable isotope signatures of five species of Silurian and Devonian deep-water, ahermatypic rugose corals, providing new insights into isotopic fractionation effects exhibited by Palaeozoic rugosans, and possible role of diagenetic processes in modifying their original isotopic signals. To minimize the influence of intraskeletal cements on the observed signatures, the analysed specimens included unusual species either devoid of large intraskeletal open spaces ('button corals': *Microcyclus*, *Palaeocyclus*), or typified by particularly thick corallite walls (*Calceola*). The corals were collected at four localities in the Holy Cross Mountains (Poland), Mader Basin (Morocco) and on Gotland (Sweden), representing distinct diagenetic histories and different styles of diagenetic alteration. To evaluate the resistance of the corallites to diagenesis, we applied various microscopic and trace element preservation tests. Distinct differences between isotopic compositions of the least-altered and most-altered skeleton portions emphasise a critical role of material selection for geochemical studies of Palaeozoic corals. The least-altered parts of the specimens show marine or near-marine stable isotope signals and lack positive correlation between δ^13^C and δ^18^O. In terms of isotopic fractionation mechanisms, Palaeozoic rugosans must have differed considerably from modern deep-water scleractinians, typified by significant depletion in both ^18^O and ^13^C, and pronounced δ^13^C-δ^18^O co-variance. The fractionation effects exhibited by rugosans seem similar rather to the minor isotopic effects typical of modern non-scleractinian corals (octocorals and hydrocorals). The results of the present study add to growing evidence for significant differences between Scleractinia and Rugosa, and agree with recent studies indicating that calcification mechanisms developed independently in these two groups of cnidarians. Consequently, particular caution is needed in using scleractinians as analogues in isotopic studies of extinct coral lineages. Answering some of the pertinent palaeoecological questions, such as that of the possibility of photosymbiosis in Palaeozoic corals, may not be possible based on stable isotope data.

## Introduction

Stable isotope analyses of scleractinian corals have been successfully applied to address various types of palaeoenvironmental and palaeoecological questions (e.g., [1–5]). Application of stable isotope signatures of extinct coral groups in palaeoceanographic and palaeoecological studies remains, however, problematic and poses a considerable challenge. On one hand, the use of coral skeletons as palaeoenvironmental archives is very attractive, because, by analogy with Recent forms, one may be able to identify seasonal or even sub-seasonal patterns of skeleton development (e.g., [2,4]). Moreover, as they typically form more rapidly than many inorganic marine precipitates, biogenic materials are less prone to significant time averaging of their geochemical signals. Finally, in some sedimentary sequences corals may constitute the most widely available materials suitable for combined oxygen and carbon isotope investigations. On the other hand, however, translating isotopic data into palaeoenvironmental or palaeoecological information is often hampered by isotopic fractionation processes imposed by organisms on their skeletal tissues, so-called 'vital effects', the magnitude of which may vary significantly and be group- or even species-specific [6–11]. Due to the presence of intraskeletal organic matrix and being precipitated out of isotopic equilibrium with ambient seawater, coral carbonate may be also more susceptible to diagenetic alteration than surrounding inorganic precipitates, and in the case of extinct taxa the degree of this alteration may be difficult to determine (cf., [1,12]).

Despite these difficulties, several authors attempted deciphering the original stable isotope signatures of two extinct groups of Palaeozoic corals, Rugosa [13–16] and Tabulata [17]. These investigations gave various and, sometimes, disparate results, and different emphasis was put in each approach on documenting the extent of secondary diagenetic alteration. In the most recent study, Zapalski [17] went even as far as to use the isotope ratios to argue for the presence of photosymbionts in tabulate corals. However, besides generally poor understanding of possible non-equilibrium isotopic fractionation mechanisms in Palaeozoic corals, assessment of a potential diagenetic alteration of the primary signals remains the major problem. Consequently, the magnitude and nature of non-equilibrium isotopic fractionation during skeleton secretion remain largely unknown for Palaeozoic corals.

While some workers advocated a high preservation potential of Palaeozoic coral skeletons due to their calcitic—and not aragonitic, as in modern scleractinians—primary mineralogy (e.g., [17]), Palaeozoic corals preserved in truly pristine condition appear exceptionally rare [18,19]. Unlike the most diagenetically-resistant low-Mg calcitic organisms, such as brachiopods, rugose coral skeletons contained probably up to 5–8 mole% MgCO_3_ ('intermediate Mg calcite') [19–21], thus being potentially susceptible to some alteration during diagenetic Mg removal. Indeed, clear indications of recrystallisation are typically found in rugosan skeletons [22–25], and even some of the fibrous features once regarded as primary, such as zigzag structures, are now commonly interpreted as an effect of diagenetic restructuring [26]. Likewise, it was proposed that all Palaeozoic rugosans were affected by diagenetic recrystallisation to some extent [18]. Last but not least, precipitation of cements in open intraskeletal pores, which in most coral species occupy more space within a corallite than the skeletal elements themselves, constitutes a serious problem when sampling for stable isotope analysis. Sampling cements together with skeletal tissues may noticeably shift the isotope ratios of studied materials (e.g., [1,12,13,15,27,28]), and early syntaxial cements may be very difficult to distinguish from the original skeleton [5,24,28–30]. Altogether, this warrants particular care in selecting specimens and assessing their preservation state in stable isotope investigations of Palaeozoic calcifying cnidarians.

The present study aims to improve our understanding of biotic fractionation effects exhibited by Palaeozoic rugose corals, as well as the influence of diagenesis on carbon and oxygen isotope signals recorded in their skeletons. This is the first study to perform a number of measurements within single rugose coral specimens, as it is done for modern corals, to test for possible correlations between δ^13^C and δ^18^O values, which might be indicative of kinetic non-equilibrium fractionation. We investigate selectively coral species virtually devoid of secondary intraskeletal cements, and compare data from different localities with distinct diagenetic histories. The majority of the specimens comes from the Devonian, for which precise stable isotope curves are available (e.g., [31]), hence enabling reliable comparison between the measured signals and isotopic composition of contemporaneous seawater. To limit the possible influence of symbiont-related vital effects and environmental variability during life of a coral, all the studied species represented moderate- to deep-water, solitary forms inferred to be non-photosymbiotic. As indicated by combined transmitted light, cathodoluminescence, scanning electron microscope and trace element analyses, the investigated corals experienced different styles of diagenetic alteration and associated possible modifications of the original isotopic signals. The study documents stable isotope patterns very different than those found in modern scleractinian corals, posing important questions as to the origin of these differences and their implications for other geochemical studies on extinct coral groups.

## Material

**Fig 1:**
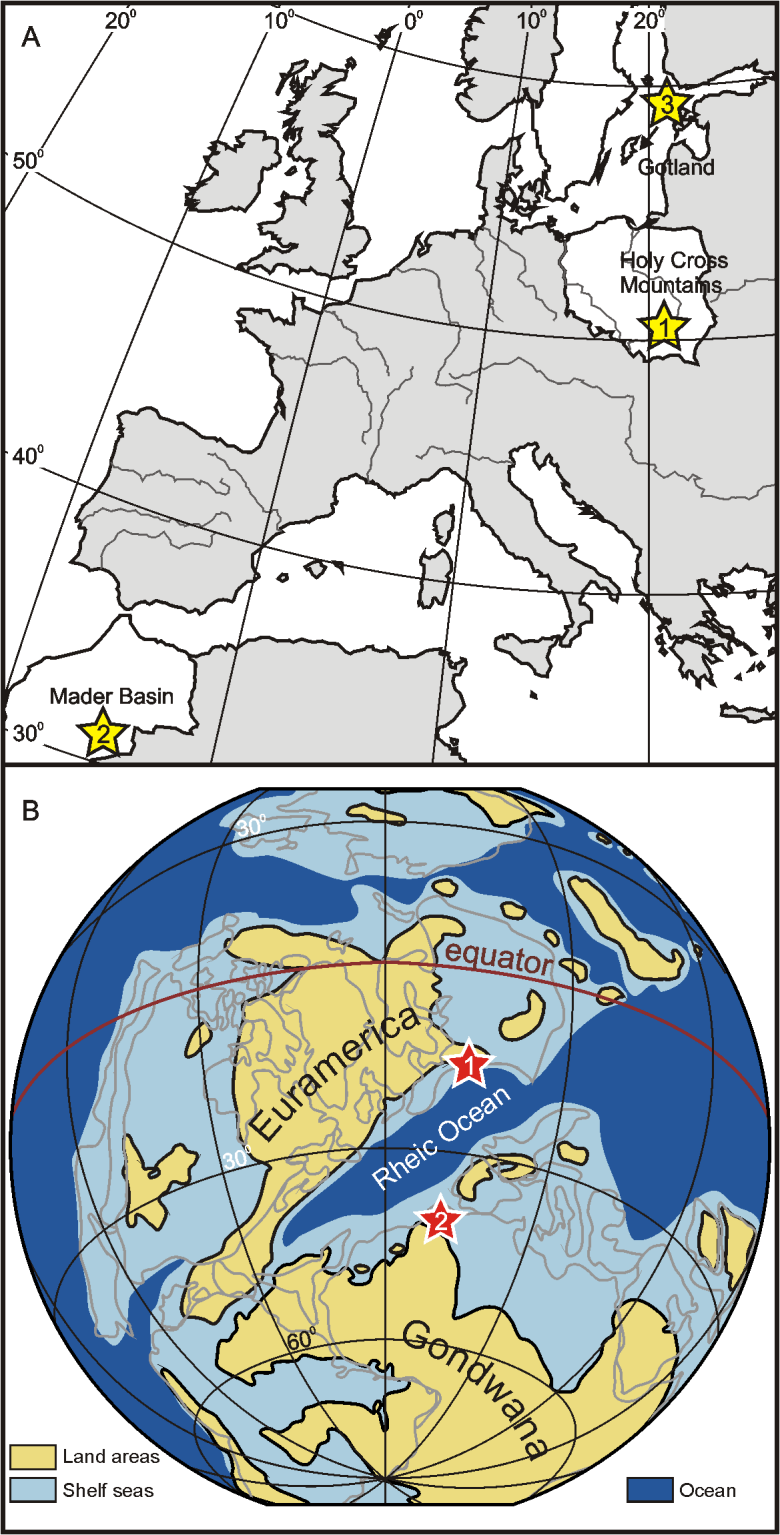
Location and palaeogeographic position of the collection areas. **A.** Locality map showing the sites where the investigated rugose corals were collected. 1: Skały, Łysogóry region of the Holy Cross Mountains, Poland; 2: Madène el Mrakib and Aferdou el Mrakib, Mader Basin, Morocco; 3: Visby, Gotland, Sweden. **B.** Middle Devonian palaeogeographic reconstruction (modified from [32]) with indicated inferred positions of the Holy Cross Mountains (1) and Mader Basin (2; Madène el Mrakib and Aferdou el Mrakib localities).

**Fig 2:**
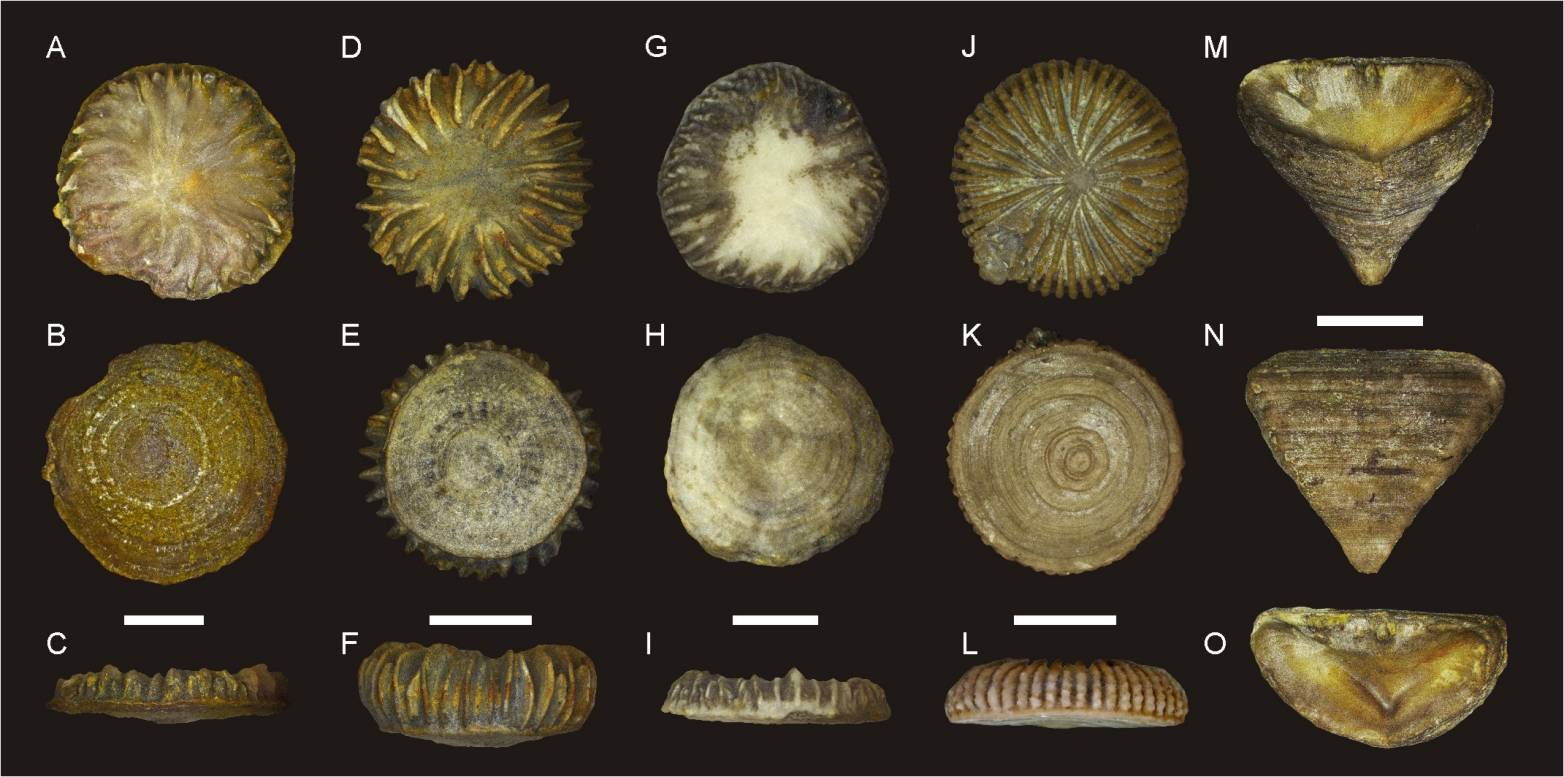
Coral specimens analysed in the present study. **A-C**: *Microcyclus praecox*, upper Eifelian, Holy Cross Mountains (Poland), UAM Tc/B/SK/20. **D-F**: *Microcyclus roberti*, lower Givetian, Madène el Mrakib (Morocco), UAM Tc/B/MM/01. **G-I**: *Microcyclus tortuosus*, lower Givetian, Aferdou el Mrakib (Morocco), UAM Tc/B/AM/01. **J-L**: *Palaeocyclus porpitus*, Telychian, Gotland (Sweden), UAM Tc/B/GO/01. **M-O**: *Calceola sandalina*, upper Eifelian, Holy Cross Mountains (Poland), UAM Tc/B/SK/50. Scale bars for A-C, D-F, G-I and J-L are 5 mm and for M-O is 1 cm.

The investigated material comprises five specimens of Silurian and Devonian rugose corals, representing five species from three different palaeogeographical settings (Figs 1, 2; S1 Fig; Table 1). The studied species either represent unusual forms that secreted very dense, compact skeletons entirely devoid of large intraskeletal pores (i.e., intertabular and interdissepimental spaces; *Microcyclus*, *Palaeocyclus*), or formed particularly thick corallite walls (*Calceola*), thus minimizing the contribution of intraskeletal cements to the measured isotope ratios. Four specimens are from Middle Devonian deposits. A single coral from the lower Silurian of Gotland (*Palaeocyclus porpitus*) has been included in the study for the sake of comparison, because it constitutes a specimen with the most obvious indications of diagenetic recrystallisation.

**Table 1:** Taxonomy, localities and the most important textural features of the studied coral specimens.

Genus	Species	Spec. #	Age	Locality	Textural preservation	Luminescence pattern	Figures
*Microcyclus*	*praecox*	UAM Tc/B/SK/20	uppermost Eifelian (*ensensis Z*one?)	Skały, Holy Cross Mountains, Poland (50°53&rsquo;43.76'' N, 21°09&rsquo;32.53'' E)	Lower part with numerous ghosts of original structures (septae with centres of calcification, interseptal stereome); upper part strongly recrystallized	Lower part dully (red) luminescent, upper part brightly luminescent	2A-C, 3A-F, 6A-B
*Microcyclus*	*roberti*	UAM Tc/B/MM/01	lowermost Givetian (*hemiansatus* Zone)	Madène el Mrakib, Mader Basin, Morocco (30°44&rsquo;03.11'' N, 4°42&rsquo;15.52'' W)	Central part with recognizable former centres of calcification and interseptal stereome; outermost portions strongly recrystallized	Non-luminescent except for the outermost part (brightly luminescent)	2D-F, 3G-L, 6D-F
*Microcyclus*	*tortuosus*	UAM Tc/B/AM/01	lowermost Givetian (*hemiansatus* Zone)	Aferdou el Mrakib, Mader Basin, Morocco (30°45&rsquo;50.76'' N, 4°37&rsquo;12.87'' W)	Recrystallized with few traces of original textural elements	Dark-blue luminescent except for the outermost part and borings (brightly luminescent)	2G-I, 4A-C, 6G-I
*Palaeocyclus*	*porpitus*	UAM Tc/B/GO/01	Telychian	Visby, Gotland, Sweden (57°35&rsquo;24'' N 18°11&rsquo;01'' E)	Strongly recrystallized with a lack of recognizable original structures	Brightly (red to orange) luminescent	2J-L, 4D-F, 6M-N
*Calceola*	*sandalina*	UAM Tc/B/SK/50	lowermost Givetian (*hemiansatus* Zone)	Skały, Holy Cross Mointains, Poland (50°53&rsquo;43.76'' N, 21°09&rsquo;32.53'' E)	Inner parts with well-defined growth increments and laminar ultrastructure; outer parts recrystallized	Inner part dully luminescent, outer part red luminescent	2M-O, 4G-L 6J-L

All the specimens are housed at the Adam Mickiewicz University in Poznań (Poland). The fieldwork in the Mader Basin (Morocco) was authorised by the Moroccan Ministry of Energy, Mining, Water and Environment (permit No. 604/14/DDM/DTG/SP).

### 'Button corals'

Three specimens of *Microcyclus* (representative of three closely related species, *M*. *praecox*, *M*. *roberti* and *M*. *tortuosus*) and one specimen of a genus *Palaeocyclus* (*P*. *porpitus*) represent an unusual, so-called 'button coral' type of corallite morphology. Such corals are characterized by a discoid shape, flat and circular epitecate surface, plano-convex corallite profile with pronounced, vertically arranged septal plates, and a lack of a typical calice. Based on analogies with modern Scleractinia, this corallite architecture is usually interpreted as a manifestation of a free-living mode of life [33–35]. Unlike most other rugosans, ‘button corals’ did not produce either tabulae or dissepiments, leaving no empty spaces available for diagenetic cement precipitation. The group of Palaeozoic 'button corals' seems to be polyphyletic, and the discoid morphologies must have developed independently in several lineages of Rugosa. *Palaeocyclus* and *Microcyclus* genera are not closely related and belong to two different families, Palaeocyclidae and Hadrophyllidae, respectively [22] (see also [36] and [37] for discussions on the taxonomy of the so-called *Microcyclus* group). 'Button corals' are most commonly found in marls and shales deposited in relatively deep-water settings, where the discoid corallite shape enabled living on unconsolidated, muddy substrates by preventing a coral from sinking into fine-grained sediment [35].

The specimen of *M*. *praecox* was collected at Skały section in the Łysogóry region of the Holy Cross Mountains, central Poland (see [38] and Fig 1 therein for the locality map and a geological section). *Microcyclus* corals occur here in Middle Devonian, clay-rich deposits with marl and limestone intercalations, so-called Skały Beds, within a distinct, siliciclastic-dominated horizon referred to as '*Microcyclus* shales' [39]. Dzik [40] assigned these shales to the latest Eifelian on the basis of tentaculite and conodont faunas. The Skały Beds, well-known for their rich and well-preserved fauna, reflect sedimentation in a relatively deep-water, intrashelf setting (see [41] and references therein).

The specimens of *M*. *roberti* and *M*. *tortuosus* come from the southern part of the Mader Basin, located in the eastern Anti-Atlas, Morocco (see [42] and Figs 5, 10 and 31 therein for the locality map and geological sections). During the Devonian, the area constituted an isolated, intrashelf basin that developed due to gradual disintegration of the north-western margin of Gondwana (e.g., [42]). Both specimens come from marly to shaly sediments situated in the lower part of the carbonate successions of the Mader Basin, dated to the earliest Givetian (*hemiansatus* conodont Zone; see [42] for a detailed stratigraphic framework). The corals are representative of two proliferous *Microcyclus* occurrences in the area. The specimen of *M*. *tortuosus* was collected at the base of a huge, reef-like buildup, so-called Aferdou el Mrakib, which developed during the Givetian in a relatively deep-water (most likely aphotic zone) carbonate ramp setting (see [42–44] for a detailed interpretation of the sedimentary environment). Here, the corals occur in the strata directly underlying the mound deposits and are contemporaneous with rich brachiopod assemblages described by Kaufmann [42] and Franchi et al. [45]. The specimen of *M*. *roberti* was collected at Madène el Mrakib, a locality situated 7 km west of Aferdou el Mrakib, in the south-westernmost part of the basin. *Microcyclus* corallites are found here in fine-grained strata underlying small carbonate mound deposits, within a shallowing-upward succession interpreted to have formed on the ramp, in somewhat more proximal position than that of the Aferdou el Mrakib [42,44,46].

A single specimen of *P*. *porpitus* was collected on Gotland (Sweden). During the early Palaeozoic, the area was located on a low-latitude carbonate platform stretching along the southern margin of Baltica. The Silurian succession in Gotland developed in parallel with slowly increasing subsidence as the Baltic passive continental margin transformed into a foreland basin during the closure of the Tornquist and Iapetus oceans throughout the Silurian [47]. *P*. *porpitus* is an index taxon for the Lower Visby Beds, which span late Telychian (Llandovery) to early Sheinwoodian (Wenlock) strata (*Pterospathodus amorphognathoides* to Lower *P*. *procerus* conodont Zones) [48]. The Lower Visby Beds are developed as alternations of unfossiliferous 2–5 cm-thick, wavy-bedded to nodular argillaceous limestones (predominantly mudstones) and ca. 10 cm-thick marls with sparse biostromes formed by the tabulate coral *Halysites* [49]. They are interpreted as deposited in an outer shelf environment below the photic zone [50]. The specimen used in this study was collected from the float in the vicinity of Ygne at the western coast of Gotland.

### Calceola sandalina

Except for the discoid corals, a single specimen of a peculiar, operculate species *Calceola sandalina* was selected for stable isotope investigations. *C*. *sandalina* secreted elongated, hemispheral corallites with deep calices having neither tabulae nor dissepiments, and terminated with an operculum (see [22] and [51] for detailed description of the *Calceola* morphology). The walls of the corallites are typically thick and can show well-defined growth increments. Most authors attribute the morphology of the species to an unusual life position of the coral, which probably lay freely with its flat side on the seafloor (e.g., [51,52]). *C*. *sandalina* inhabited soft-bottom substrates and is typically found in marly and shaly sediments deposited in moderate- to deep-water settings. The specimen investigated in the present study comes from the same horizon as the specimen of *M*. *praecox*, i.e., the uppermost Eifelian '*Microcyclus* shales' of the Skały section in the Holy Cross Mountains (see above for more details).

## Methods

All the studied corals were physically cleaned with a sandblaster and an ultrasonic cleaner, air dried and photo-documented. Then, the specimens were embedded in epoxy resin in a vacuum chamber, and cut transversally to prepare standard petrographic thin sections. The thin sections were subjected to transmitted, plane- and cross-polarized light microscopic investigations, and analysed for cathodoluminescence (CL) with a Technosyn 8200 MK II luminoscope system operating under 14–16-kV accelerating voltage and 200–300 μA beam current. Subsequently, the sections were etched with 0.1 M HCl for 20–30 s, rinsed with distilled water, air dried, sputter coated with gold and analysed under 5–20-kV accelerating voltage with a scanning electron microscope (SEM) Tescan Vega\\XMU at GeoZentrum Nordbayern, Friedrich-Alexander University of Erlangen-Nürnberg (Germany).

Elemental analyses were performed on graphite-coated polished thin sections with a Cameca SX-100 electron microprobe operating in the WDS mode under 15 kV accelerating voltage, 20 nA beam current and 10 μm spot diameter at the Inter-Institute Analytical Complex for Minerals and Synthetic Substances, Electron Microprobe Laboratory, University of Warsaw (Poland). Six elements (Ca, Mg, Mn, Fe, Ba, and Sr) were analysed in each sampling spot. The following standards, analytical lines and crystals were used: diopside—Mg (Kα, TAP), Ca (Kα, PET); rhodonite—Mn (Kα, LPET); hematite—Fe (Kα, LIF); barite—Ba (Lα, LPET); and celestine—Sr (Lα, TAP). A ZAF matrix correction was applied to all analyses. The average reproducibility (1σ) was better than ±0.04% for Mg, 0.55% for Ca, 0.02% for Mn, 0.04% for Fe, 0.06% for Ba, and 0.07% for Sr. Detection limits for each analysis are given in Table 2; Ba concentrations were always below detection limits of 443–517 ppm.

**Table 2:** Elemental data for the studied rugose coral specimens, including the least-altered (LA) and strongly altered (A) corallite portions identified based on microfacies investigations.

Sample #	Pres.	Ca	Mg	Mn	Fe	Sr
*M*. *praecox*, *Holy Cross Mountains*						
SK-1	LA	39.22	3195	329	3941	594
SK-2	LA	39.09	3331	462	3155	516
SK-3	LA	39.49	2990	479	2779	423
SK-4	LA	38.95	4616	504	2621	496
SK-5	LA	39.55	2833	417	3444	345
SK-6	LA	39.05	3093	428	2897	669
SK-A-1	A	39.41	3764	565	1681	585
SK-A-2	A	39.73	3657	396	314	528
Det. limits		≤0.05	≤216	≤133	≤356	≤291
*M*. *roberti*, *Madène el Mrakib*						
MM-1	LA	39.57	6212	bdl	bdl	928
MM-2	LA	39.23	6149	bdl	bdl	934
MM-3	LA	39.70	6452	bdl	bdl	777
MM-4	LA	39.08	6683	bdl	bdl	1027
MM-5	LA	39.00	6764	bdl	bdl	690
MM-6	LA	39.11	5759	bdl	bdl	1081
MM-A-1	A	39.28	6932	bdl	346	899
MM-A-2	A	39.70	6270	bdl	bdl	853
Det. limits		≤0.05	≤219	≤136	≤377	≤312
*M*. *tortuosus*, *Aferdou el Mrakib*						
AM-1	LA	39.45	6023	bdl	bdl	438
AM-2	LA	39.95	5226	bdl	bdl	492
AM-3	LA	39.34	4909	bdl	bdl	412
AM-4	LA	39.83	2797	bdl	bdl	bdl
AM-5	LA	39.59	4516	bdl	bdl	536
AM-A-1	A	40.15	3723	bdl	bdl	683
Det. limits		≤0.05	≤224	≤136	≤376	≤305
*P*. *porpitus*, *Gotland*						
GO-1		39.23	3724	446	1795	498
GO-2		39.34	4545	424	944	756
GO-3		39.26	4716	336	1051	882
GO-4		38.99	3783	454	707	699
Det. limits		≤0.05	≤217	≤136	≤375	≤310
*C*. *sandalina*, *Holy Cross Mountains*						
CL-1	LA	39.668	5048	bdl	423	704
CL-2	LA	39.0813	6115	bdl	1502	449
CL-3	LA	39.0991	4150	bdl	1363	bdl
CL-4	LA	39.0794	5449	bdl	bdl	936
CL-5	LA	39.5597	5565	bdl	521	907
CL-6	LA	38.6275	6969	bdl	bdl	926
CL-A-1	A	39.3411	4959	154	2048	442
CL-A-1	A	39.104	4141	145	1575	bdl
Det. limits		≤0.05	≤228	≤137	≤381	≤312

Ca values in weight%; Mg, Mn, Fe, Sr values in ppm. Abbreviations: Pres.—preservation state; LA—least-altered parts of the corallites; A—strongly altered parts of the corallites; det. limits—detection limits; bdl—below detection limit.

Powder samples for carbon and oxygen isotope measurements were taken from slabbed coral surfaces using a Merchantek MicroMill sampling device. The microdrilling sampling technique, commonly used in investigations of modern corals (e.g., [53, 54]), is here applied for the first time in studies of Palaeozoic coral skeletons. The use of a MicroMill as such is not necessary to obtain reliable stable isotope signatures of corallites, as depletion in the heavy isotopes and correlation between δ^13^C and δ^18^O, if present, should be detectable with randomly selected, relatively large samples collected using a classic, hand-held microdrill [11,55]. Nonetheless, the advantage of micromilling is that it assures precise control on the characteristics of the material sampled and minimal depth of the sampling tracks, which is of importance given the presence of bright-luminescent zones indicating secondary alteration, and cement-filled borings within the studied specimens. Only in the case of the *P*. *porpitus* corallite, which shows the highest level of diagenetic textural homogenization, samples were taken with a conventional microdrill.

The sampling tracks were chosen based on previous transmitted-light, cathodoluminescence and scanning electron microscope observations so that to include samples of both least-recrystallised and most-recrystallised (usually outermost) parts of the skeletons. Particular care was exercised to avoid sampling local skeleton inhomogeneities, such as borings. Except for the strongly recrystallised specimen of *P*. *porpitus*, 15 to 16 samples of the least-altered material, and 2–3 samples of the obviously altered portions were taken from each coral; according to Smith et al. [55], 9 samples is sufficient to reliably show a correlation between δ^13^C and δ^18^O values in contemporary corals. Only in the case of *C*. *sandalina*, the presence of well-defined growth increments enabled sampling a real time series; in the skeletons of *Microcyclus* and *Palaeocyclus* corals, the original growth increments could not be precisely traced, and the sampling tracks mostly cut them diagonally. This results in some extent of time averaging for the button coral samples; given the small size of the specimens and the lack of visible primary growth lines, such time averaging is, however, inevitable regardless of the sampling technique used.

Stable isotope composition of the carbonate powders was determined at the Stable Isotope Laboratory of GeoZentrum Nordbayern, Friedrich-Alexander University of Erlangen-Nürnberg. CO_2_ was released for analysis by treating the samples with 103% H_3_PO_4_ at 70°C with an automated Gasbench II device and analysed for carbon and oxygen isotopes using a ThermoFisher Delta V Plus mass spectrometer. All isotope ratios are given in standard δ notation in ‰ relative to the V-PDB standard. Accuracy of the analyses was monitored by measurements of laboratory standards calibrated to international standards NBS19 (δ^13^C = 1.95‰, δ^18^O = −2.20‰) and LSVEC (δ^13^C = −46.6‰, δ^18^O = −26.7‰). The average reproducibility (1σ) was ±0.07‰ for δ^13^C and ±0.08‰ for δ^18^O.

## Results

### Microstructure

The studied corals show different levels of secondary skeleton restructuring and diagenetic alteration. The most important of textural and cathodoluminescence observations are summarized in Table 1.

**Fig 3:**
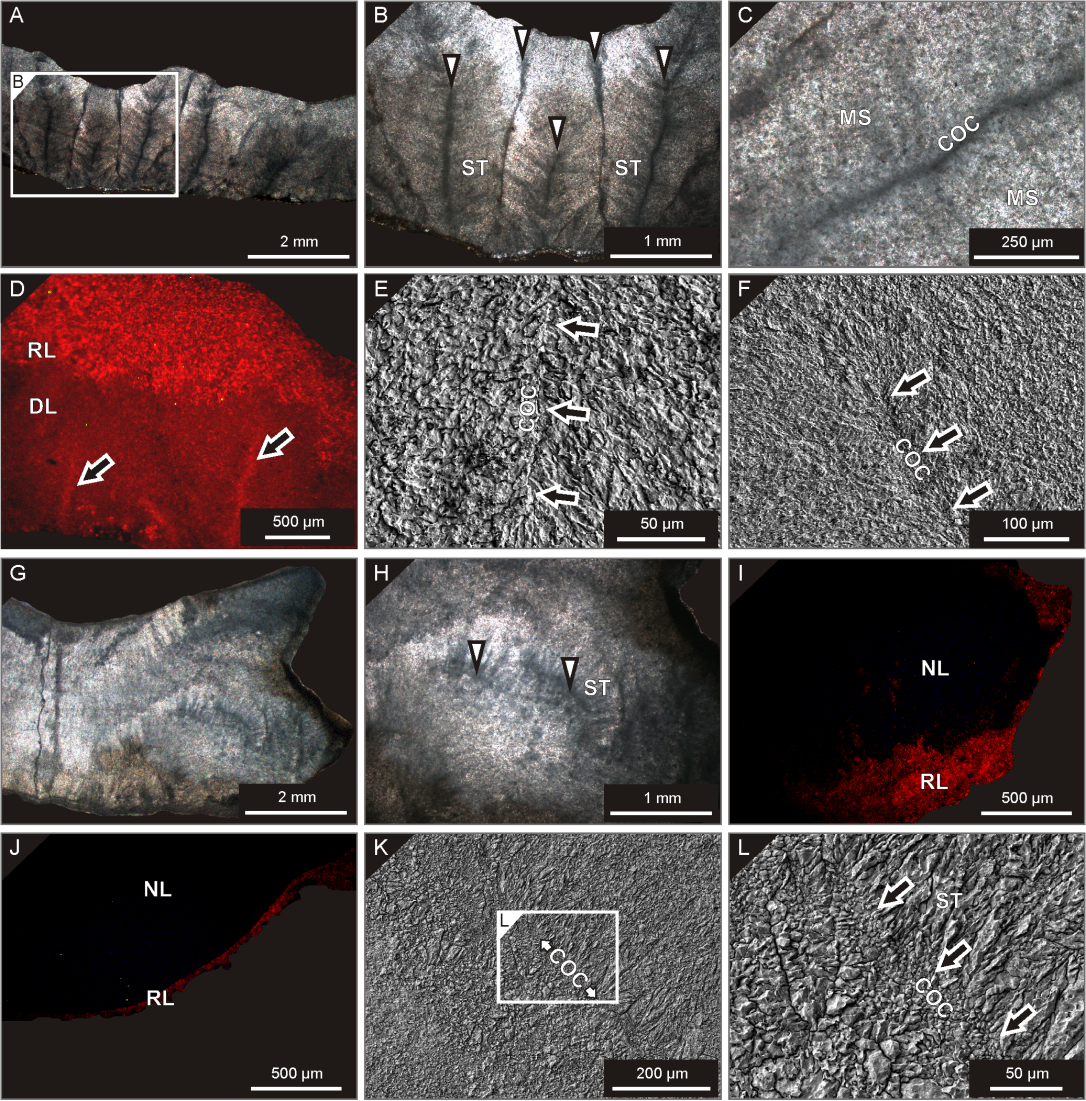
Micro- and ultrastructure of the studied coral specimens. **A-F**: *Microcyclus praecox*, upper Eifelian, Holy Cross Mountains (Poland), UAM Tc/B/SK/20. **A-B.** Transmitted-light photomicrographs showing clearly distribution of septa (with 'dark lines' representing former centres of calcification; arrows) and fibrous interseptal stereome (ST). **C.** Close-up of a former centre of calcification (COC); note the presence of microspar to small-spar crystal mosaic (MS) cutting across the primary structural elements. **D.** CL view; note clear difference in the luminescence pattern between lower (dully luminescent, DL) and uppermost (red- to orange-luminescent, RL) parts of the corallite. Location of former centres of calcification is underlined by their bright luminescence (arrows). **E-F.** SEM photomicrographs of former centres of calcification (COC, arrows). **G-L**: *Microcyclus roberti*, lower Givetian, Madène el Mrakib (Morocco), UAM Tc/B/MM/01. **G-H.** Transmitted-light views showing traces of the original skeletal elements mostly preserved as ghosts within the irregular calcite crystal mosaic. Former centres of calcification (cut diagonally) with recognizable thickening deposits of fibrous stereome (ST) are indicated with arrows. **I-J.** CL patterns with distinct luminescence responses of a central, relatively well-preserved portion of the corallite (non-luminescent, NL), alteration rims surrounding the original skeleton, as well as diagenetic cement encrusting the corallite surface (both red-luminescent, RL; see a lower right part of G for a transmitted-light view of I). **K-L.** SEM photomicrographs illustrating a well-defined former centre of calcification (COC, arrows) with still recognizable original fibrous texture of interseptal stereome (ST).

Among the button coral specimens, *M*. *praecox* from the Holy Cross Mountains represents the one with best visible primary microstructural features when observed under transmitted-light. Former centres of calcification ('dark lines') are distinctly defined, and original stereome fibres radiating from the axial planes of septa are still easily recognizable (Fig 3A and 3B). As revealed by cross-polarized light observations, present boundaries of inclusion-rich calcite crystals usually cut across the outlines of the original skeletal features (Fig 3B and 3C), indicating that most of these structures are preserved as ghosts, rather than pristine skeletal elements themselves. SEM investigations show a generally fine (mostly in the microspar to small spar range) calcite crystal mosaic bearing little information on the original skeletal texture, though former centres of calcification are still noticeable (Fig 3E and 3F). Cathodoluminescence analyses reveal pronounced, red to orange luminescence of the upper portions of the corallites consisting of small, irregular calcite crystals, and dull to red luminescence of the lower part of the skeleton. Only the former centres of calcification stand out against the background due to their more distinct, orange luminescence (Fig 3D).

**Fig 4:**
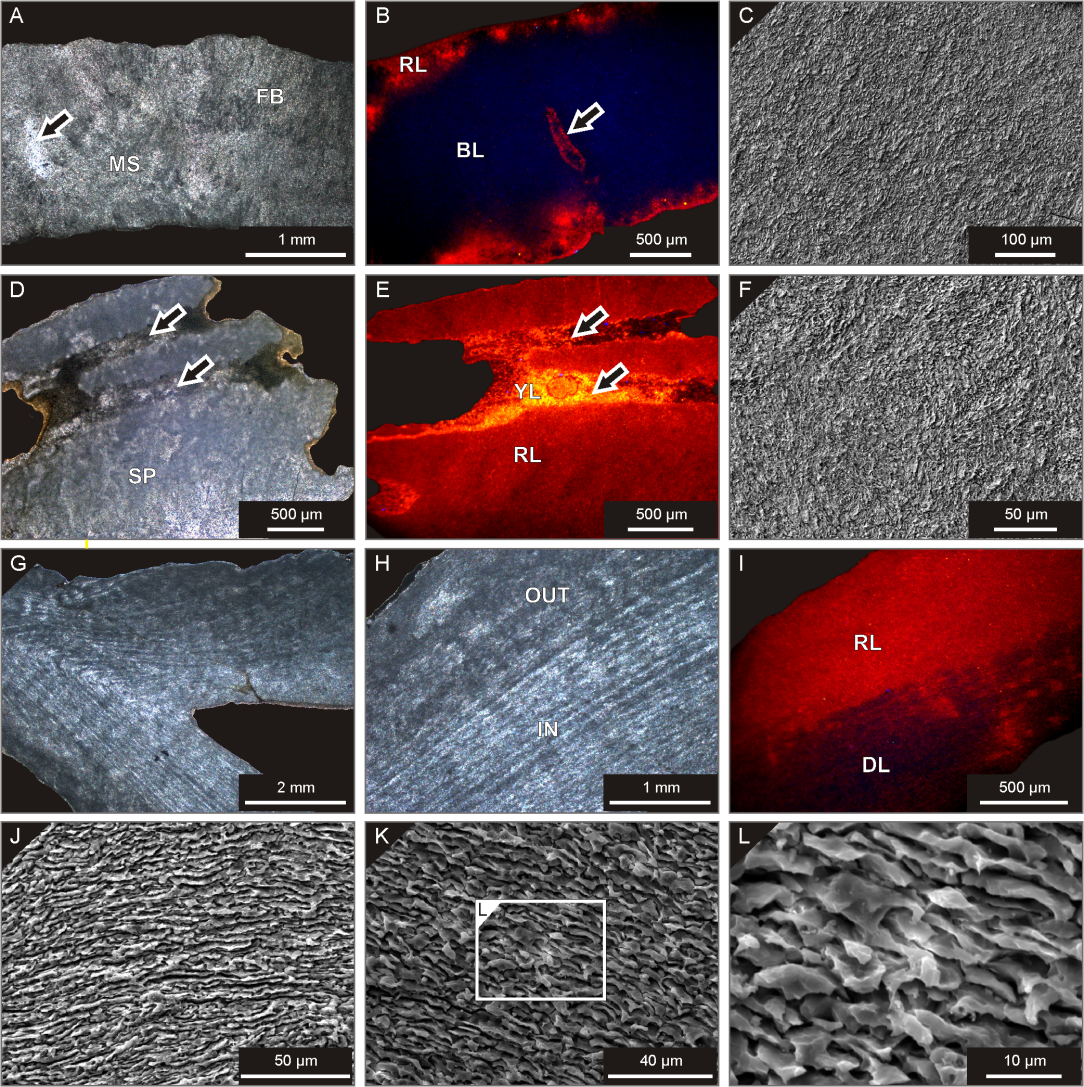
Micro- and ultrastructure of the studied coral specimens. **A-C**: *Microcyclus tortuosus*, lower Givetian, Aferdou el Mrakib (Morocco), UAM Tc/B/AM/01. **A.** Transmitted-light view; the corallite has been mostly recrystallised to a uniform, fine calcite crystal mosaic (MS) with rare ghosts of the original fibrous texture (FB). Note the presence of a cement-filled boring (arrow). **B.** CL pattern showing the dark blue-luminescent (BL) centre of the corallite and its red-luminescent outer parts (RL). Note also a contrasting luminescence pattern of a boring (arrow; see A for a transmitted-light view). **C.** SEM view showing invariably small (microspar to small spar), irregular calcite crystals indicative of strong recrystallisation of the skeleton. **D-F**: *Palaeocyclus porpitus*, Telychian, Gotland (Sweden), UAM Tc/B/GO/01. **D.** Transmitted-light photomicrograph; note recrystallisation of the original skeletal material to small sparry calcite crystals (SP) and the entire lack of visible original microstructural features. The interseptal spaces were partially occluded by early diagenetic cements (arrows). **E.** CL view; the specimen shows uniform red luminescence (RL) with only interseptal spaces filled by yellow to orange-luminescent cements (YL, arrows—see D for a transmitted-light view). **F.** SEM photomicrograph illustrating complete transformation of the original corallite microstructure into a uniform, fine calcite crystal mosaic. **G-L**: *Calceola sandalina*, upper Eifelian, Holy Cross Mountains (Poland), UAM Tc/B/SK/50. **G-H.** Transmitted-light views showing well-defined original growth increments in the inner part (IN) of the corallite walls, and more advanced recrystallisation of the outer (OUT) skeleton portions. **I.** CL view of a corallite portion shown in H; note distinct contrast between laminated, dull to faintly-red luminescent (DL), inner part of the wall and homogenously red-luminescent (RL) outer portion of the corallite. **J-L.** SEM photomicrographs showing well-defined lamellar ultrastructure of the corallite wall.

Of the two Moroccan representatives of *Microcyclus*, the *M*. *roberti* specimen from Madène el Mrakib still shows well-defined traces of original fibrous stereome and centres of calcification, similar to those found in *M*. *praecox*. These are mostly preserved as ghosts not coinciding with the current calcite crystal outlines (Fig 3G and 3H). SEM analyses show the presence of microspar to small spar crystal mosaic, but the sites of the original centres of calcification are still well visible (Fig 3K and 3L). The *M*. *roberti* corallite shows no luminescence in the central and upper portion of the skeleton, whereas the lower part of the corallite gives red luminescence responses (Fig 3I). A thin layer of calcitic cement rimming the corallite has a distinct red to orange luminescence (Fig 3J). A skeleton of *M*. *tortuosus* from the Aferdou el Mrakib locality, in turn, consists mostly of microspar to fine spar cloudy calcite crystals, with few remnants of the original fibrous texture (Fig 4A), no visible centres of calcification, and a very homogenous calcite crystal mosaic visible under SEM (Fig 4C). The corallite shows dark blue luminescence in its central part, and only borings and peripheral portions of the skeleton luminesce red to orange (Fig 4B).

The *P*. *porpitus* specimen represents the most advanced level of diagenetic restructuring, with its skeletal tissue invariably recrystallised to fine, irregular sparry crystals preserving no traces of pristine skeletal features, as revealed by both transmitted-light and scanning electron microscope observations (Fig 4D and 4F). In places, the presence of early diagenetic sparry cements is apparent in interseptal spaces (Fig 4D). The *P*. *porpitus* corallite shows pronounced red to orange luminescence, with early cements usually showing more distinct, orange to yellow luminescence responses with noticeable individual fine calcite crystals (Fig 4E).

The skeletal micro- and ultrastructure of *C*. *sandalina* is conspicuously different from that of the button corals. Here, distinctly defined growth increments parallel to the calice outline are well visible in longitudinal sections in the inner parts of the corallite walls (Fig 4G and 4H). The presence of lamination is even more distinct when observed with SEM; the individual laminae consist of fine (ca. 1–5 μm in thickness and 5–30 μm in length) calcite crystal flakes oriented with their long axes parallel to the laminae boundaries and forming a lamellar ultrastructure (Fig 4J–4L). The outer parts of the corallite walls show less pronounced lamination and are made up predominantly of irregular crystal mosaic (Fig 4G and 4H). The difference between the well-layered, inner portion of the corallite and the more recrystallised, outer part is reflected in distinct cathodoluminescence patterns of these two portions of the wall, with the former typified mostly by dull luminescence with some intercalations of red-luminescent laminae, and the latter by distinct red luminescence with recognizable irregular, microspar to small spar calcite crystals (Fig 4I).

### Elemental concentrations

Concentrations of the analysed major (Ca) and minor (Mg, Mn, Fe, Sr) elements are given in Table 2. In general terms, the elemental data concur with the microscopic investigations, pointing to differences in preservation between inner and outer portions of the corallites. The non- to dull-luminescent parts of the skeletons are characterized typically by lower Mn and Fe contents (<150 ppm for Mn and <1600 ppm for Fe) than the bright-luminescent areas, in which Mn and Fe concentrations up to 565 and 2038 ppm, respectively, were measured. The only exception is the *M*. *praecox* corallite from the Holy Cross Mountains, in which the dull luminescence results from considerably higher Fe contents (from ca. 2800 to nearly 4000 ppm) overprinting the effect of relatively high Mn concentrations. In two *Microcyclus* specimens from Morocco, which show no or bluish luminescence, both Mn and Fe concentrations were always found below detection limits (≤137 ppm for Mn and ≤384 ppm for Fe; unaltered, marine-equilibrated calcites usually exhibit Mn and Fe concentrations from 0 to 200 and 0 to 600 ppm, respectively (e.g., [15, 56–58]). Dissimilarities in the distribution of Mg and Sr in the least-altered and conspicuously altered portions of the corallites are less clearly defined, yet distinct differences can be found in Mg and Sr concentrations among different specimens. The highest contents of both Mg and Sr (more than 6700 ppm and 1000 ppm, respectively) are observed in the non-luminescent *M*. *roberti* corallite from Madène el Mrakib. The lowest Mg concentrations (3343 ppm on average), in turn, are displayed by the *M*. *praecox* corallite, whereas the strongest depletion in Sr (≤536 ppm) typifies the bluish-luminescent corallite of *M*. *tortuosus* from Aferdou el Mrakib.

### Stable isotopes

**Fig 5:**
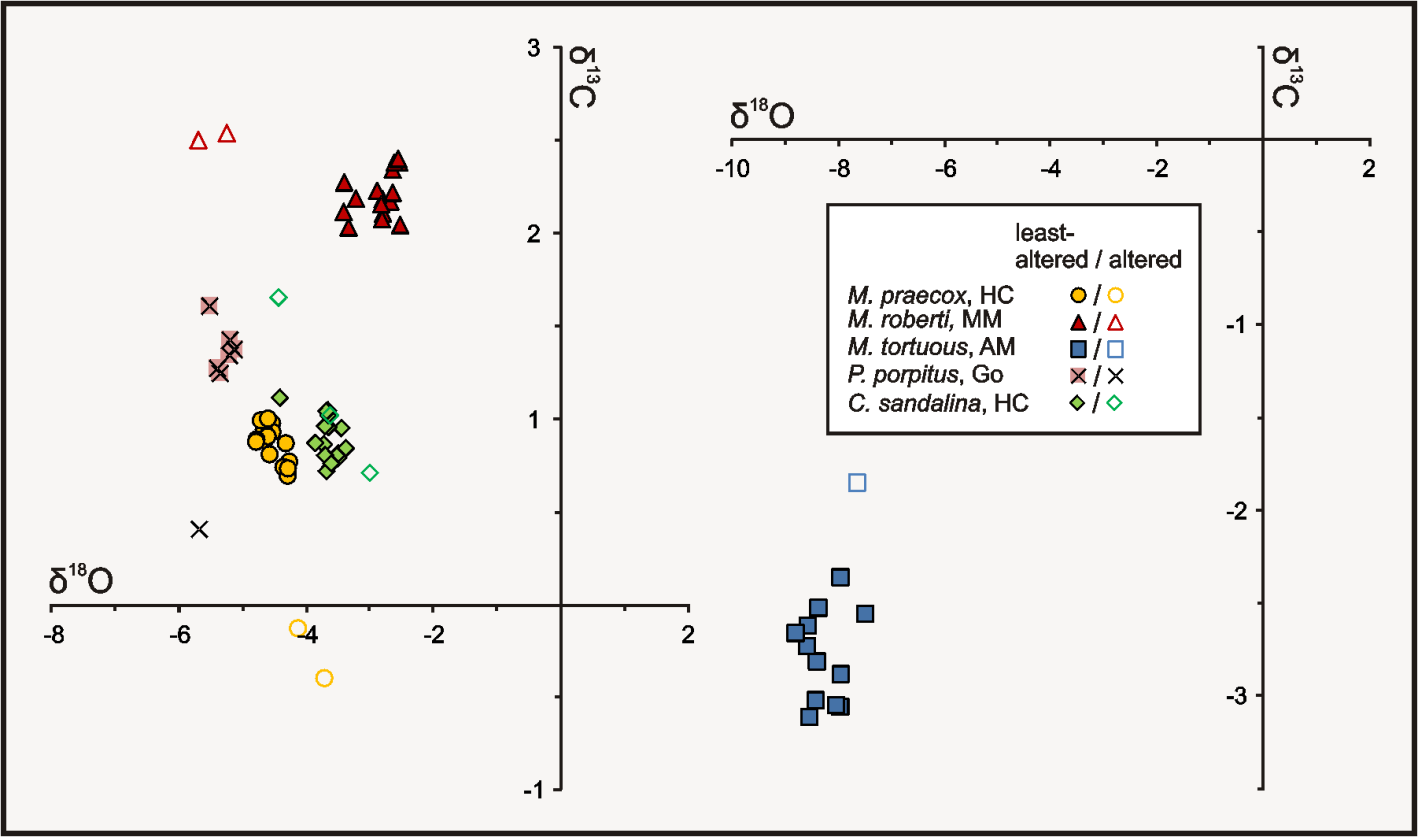
Carbon vs. oxygen isotope cross-plots for the studied corallites. Note that the samples selected as the most obviously altered ones fall mostly clearly outside the fields defined by the δ^13^C and δ^18^O values of the least-altered portions of the corallites. All results are shown in ‰ V-PDB. Abbreviations: HC—Holy Cross Mountains; MM—Madène el Mrakib; AM—Aferdou el Mrakib; Go—Gotland.

**Fig 6:**
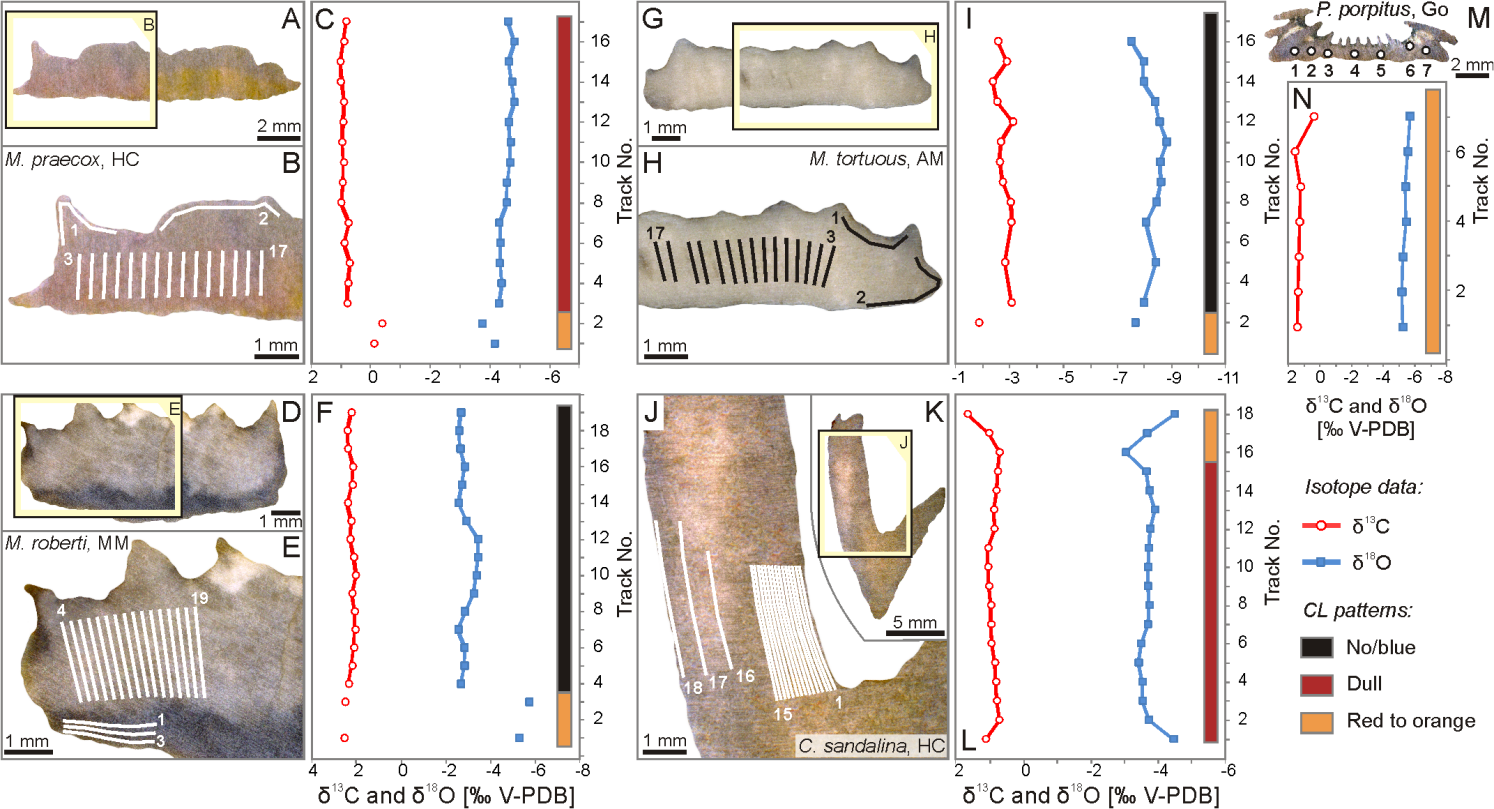
Distribution of sampling tracks, and the δ^13^C and δ^18^O values found in the individual tracks within the studied coral specimen. Tracks forming continuous isotopic transects are connected with continuous lines. Note the relationship between the occurrence of anomalous isotopic values and distinct luminescence patterns of the sampled carbonates. **A-C**: *Microcyclus praecox*, upper Eifelian, Holy Cross Mountains (Poland), UAM Tc/B/SK/20; **D-F**: *Microcyclus roberti*, lower Givetian, Madène el Mrakib (Morocco), UAM Tc/B/MM/01; **G-I**: *Microcyclus tortuosus*, lower Givetian, Aferdou el Mrakib (Morocco), UAM Tc/B/AM/01; **J-L**: *Calceola sandalina*, upper Eifelian, Holy Cross Mountains (Poland), UAM Tc/B/SK/50; **M-N**: *Palaeocyclus porpitus*, Telychian, Gotland (Sweden), UAM Tc/B/GO/01. Abbreviations as in Fig 5.

Each of the analysed coral specimens shows somewhat different ranges in δ^13^C and/or δ^18^O (Table 3), which results in clustering of the values in five non-overlapping fields (Fig 5). No obvious trends in changes of the stable isotope composition occur along the analysed transects (Fig 6), and there is little co-variance between δ^13^C and δ^18^O values except for the single specimen of *M*. *praecox* from the Holy Cross Mountains, in which some negative correlation (r = −0.70, p = 0.004) can be noticed. In each case, strongly altered portions of corals display δ^13^C and/or δ^18^O signals distinct from those of the least-altered parts of the skeletons, and thus fall conspicuously outside the clusters defined by them.

**Table 3:** Stable isotopic composition of the least-altered (LA) and strongly altered (A) parts of the analyzed rugosan corallites.

Track #	Pres.	δ^13^C	δ^18^O
*M*. *praecox*, *Holy Cross Mountains*			
1	A	-0.12	-4.16
2	A	-0.39	-3.74
3	LA	0.78	-4.29
4	LA	0.75	-4.38
5	LA	0.70	-4.32
6	LA	0.88	-4.35
7	LA	0.74	-4.31
8	LA	0.98	-4.56
9	LA	0.94	-4.56
10	LA	0.89	-4.68
11	LA	0.95	-4.69
12	LA	0.91	-4.63
13	LA	0.90	-4.81
14	LA	1.00	-4.74
15	LA	1.01	-4.63
16	LA	0.88	-4.81
17	LA	0.82	-4.60
*M*. *roberti*, *Madène el Mrakib*			
1	A	2.55	-5.27
2	A	nd	nd
3	A	2.51	-5.72
4	LA	2.35	-2.67
5	LA	2.19	-2.83
6	LA	2.11	-2.82
7	LA	2.05	-2.55
8	LA	2.08	-2.84
9	LA	2.19	-3.25
10	LA	2.04	-3.36
11	LA	2.12	-3.44
12	LA	2.28	-3.43
13	LA	2.24	-2.91
14	LA	2.39	-2.57
15	LA	2.18	-2.70
16	LA	2.16	-2.85
17	LA	2.39	-2.64
18	LA	2.41	-2.58
19	LA	2.23	-2.67
*M*. *tortuosus*, *Aferdou el Mrakib*			
1	A	nd	nd
2	A	-1.85	-7.64
3	LA	-3.05	-7.96
4	LA	nd	nd
5	LA	-2.81	-8.40
6	LA	nd	nd
7	LA	-3.04	-8.04
8	LA	-3.02	-8.43
9	LA	-2.73	-8.59
10	LA	-2.62	-8.57
11	LA	-2.66	-8.81
12	LA	-3.11	-8.55
13	LA	-2.52	-8.38
14	LA	-2.36	-7.96
15	LA	-2.88	-7.95
16	LA	-2.55	-7.50
*P*. *porpitus*, *Gotland*			
1		1.43	-5.22
2		1.38	-5.15
3		1.35	-5.23
4		1.28	-5.42
5		1.25	-5.37
6		1.61	-5.54
7	CC	0.41	-5.70
*C*. *sandalina*, *Holy Cross Mountains*			
1	A	1.12	-4.44
2	LA	0.73	-3.71
3	LA	0.80	-3.52
4	LA	0.82	-3.52
5	LA	0.85	-3.40
6	LA	0.96	-3.47
7	LA	0.96	-3.68
8	LA	0.97	-3.73
9	LA	1.03	-3.68
10	LA	1.05	-3.68
11	LA	1.05	-3.71
12	LA	0.87	-3.75
13	LA	0.88	-3.88
14	LA	0.81	-3.73
15	LA	0.77	-3.63
16	A	1.66	-4.46
17	A	1.03	-3.65
18	A	0.72	-3.03

Abbreviations as in Table 2; nd—no data—sample quantity insufficient for reliable measurement; CC—likely contamination by cements. All results are given in ‰ V-PDB.

The least-altered portions of four of the investigated specimens have positive and little variable δ^13^C values, ranging from 0.7 to 1‰ for *M*. *praecox*, 0.7 to 1.1‰ for *C*. *sandalina* (both from the Holy Cross Mountains), 2 to 2.4‰ for *M*. *roberti* from Madène el Mrakib, and 1.3 to 1.6‰ for *P*. *porpitus* from Gotland. The only negative δ^13^C values were measured in the corallite of *M*. *tortuosus* from Aferdou el Mrakib; this specimen exhibits also the widest range of carbon isotope values (−2.4 to −3.1‰). In the *C*. *sandalina* and both Moroccan *Microcyclus* skeletons, the δ^13^C values of the most luminescent parts are somewhat higher (by up to ca. 0.5‰ for *C*. *sandalina* and *M*. *tortuosus* from Aferdou el Mrakib, and only slightly for *M*. *roberti* specimen from Madène el Mrakib) than the values of non- to dull-luminescent corallite portions. A trend towards lower δ^13^C values with increasing diagenetic alteration can be, in turn, noticed in the *M*. *praecox* specimen from the Holy Cross Mountains. For *P*. *porpitus*, the most ^13^C-depleted value represents the part of the skeleton where conspicuous intraskeletal cements were observed.

All δ^18^O values are negative and reveal higher variability than the δ^13^C signals, though in most cases the isotope ratios from individual corals show pronounced clustering. The smallest ranges in δ^18^O are observed in the two specimens from the Holy Cross Mountains (−4.3 to −4.8‰ for *M*. *praecox*, and −3.4 to −3.9‰ for *C*. *sandalina*) and *P*. *porpitus* from Gotland (−5.2 to −5.5‰). Slightly higher variability is found in the corallite of *M*. *roberti* from Madène el Mrakib (−2.6 to −3.4‰). The most variable—and the most negative—oxygen isotope signals were measured on the *M*. *tortuosus* specimen from Aferdou el Mrakib, with δ^18^O values varying by as much as 1.3‰ (−7.5 to −8.8‰), and the highest δ^18^O value being still more than 4‰ lower than the lowest value found in the contemporaneous *M*. *roberti* corallite from Madène el Mrakib. The most luminescent portions of the skeletons can be either depleted (Madène el Mrakib) or enriched (*M*. *praecox* from the Holy Cross Mountains) in ^18^O, or fall within the range defined by the remaining data (Aferdou el Mrakib). In the case of *C*. *sandalina*, there is a trend towards first enrichment in ^18^O, and then depletion in ^18^O in the red-luminescent part of the skeleton with decreasing distance towards the external corallite wall (Fig 6).

## Discussion

The investigated coral specimens experienced different diagenetic histories and represent various states of preservation of micromorphological features. Interestingly, the preservation tests carried out with different methods did not always give analogous results, thus showing the complexity of diagenetic alteration processes and necessity of simultaneously using several different proxies.

Despite these differences in the style of diagenesis, some general features are apparent from the δ^13^C and δ^18^O values. Overall, two patterns can be distinguished in the analysed data: (i) minor variability in δ^13^C and δ^18^O values, which fall within or close to the ranges characteristic of calcites precipitated in equilibrium with contemporaneous seawater, displayed by four specimens; and (ii) significant depletion in both δ^13^C and, in particular, δ^18^O, shown by the single specimen of *M*. *tortuosus* from Aferdou el Mrakib. In the following, we show that isotopic compositions of the studied corals, in combination with their preservation state and general observations on their sedimentary and diagenetic environments, point to the first of these patterns as representing primary isotopic signals of the studied rugosans. This implies that mechanisms of incorporation of carbon and oxygen isotopes in Palaeozoic rugosans differed markedly from fractionation processes imposed by modern azooxanthellate scleractinians, which show strong vital fractionation effects resulting in conspicuous depletion in both ^13^C and ^18^O (down to ca. −5‰ for δ^18^O and even −10‰ for δ^13^C) and a clear positive correlation between δ^13^C and δ^18^O values (e.g., [7,53–55,59]).

### Preservation of carbon and oxygen isotope signals in rugose corals

#### General remarks

Three observations are of crucial importance for explanation of the stable isotope signatures of the analysed corals:

1. With the exception of *M*. *tortuosus* from Aferdou el Mrakib, the carbon and oxygen isotope values observed within the corals are tightly clustered, with no distinct relationship between δ^13^C and δ^18^O and the position of sampling tracks in the relatively unaltered portions of the skeletons. Above all, no positive correlation between δ^13^C and δ^18^O is observed in any of the specimens. The low variability and lack of consistent changes of the isotopic signatures in time is evident also in the case of *C*. *sandalina*, in which the sampling tracks represent a continuous time series.
2. Despite their calcitic primary mineralogy, all the investigated rugosans show more or less pronounced indications of diagenetic alteration, yet in most cases either some pristine structures or ghosts of the original elements are still present.
3. The areas identified as the most diagenetically altered parts of the skeletons are characterized by isotopic signatures that fall consistently outside the isotopic ranges defined by the least-altered corallite portions. This emphasises the need for careful selection of not only entire specimens, but also specific portions of corallites in studies of stable isotope composition of Palaeozoic corals.

Two explanations can be invoked to account for the narrow ranges of the δ^13^C and δ^18^O values observed: (i) the signals represent the primary values acquired during coral growth, or (ii) the observed signatures originated as a result of overprinting of the primary signals during early diagenesis, when the corallites were still in contact with relatively unaltered seawater. Despite the indications of some diagenetic restructuring of the studied specimens, reflected, especially, by the recrystallisation of the majority of the corallites to fine crystal mosaic and luminescence of some of the materials studied, the first scenario provides the most coherent explanation for the patterns analysed in the present approach.

Shelf seas, such as those inhabited by the investigated Palaeozoic coral species, are today normally supersaturated with respect to both calcite and aragonite down to several hundred metres, and little diagenetic change is typically observed in carbonates on the seafloor (e.g., [60–63]). Accordingly, studies of early marine diagenetic alteration in scleractinian corals show mostly effects of cementation and bioerosion, rather than major recrystallisation of the original skeletal material except for the most organic-rich portions of the skeleton, i.e., especially, centres of calcification [5,28,30,64–67]. To induce major recrystallisation of metastable CaCO_3_ polymorphs, i.e., aragonite and Mg-calcite, a carbonate component usually needs to be buried to some depth, where progressive decomposition of organic matter results in decrease of pH in interstitial waters [63,68,69]. Under such conditions, carbonates may indeed undergo some degree of isotopic exchange with early diagenetic fluids; such early-burial recrystallisation should be, however, detectable as distinct luminescence of the recrystallised calcite, activated by incorporation of Mn(II) produced due to microbial Mn(IV) reduction. A typical situation in such a case is also that diagenetically altered carbonates show wide scatter of isotopic values (e.g., [56,58,68,70,71]), ranging from unaltered to strongly altered ones, rather than dense data clustering, as displayed by the most of the studied rugose corallites. Analogously, it seems very unlikely that some limited seafloor dissolution of skeletons similar to that observed in modern scleractinians (cf., [29,72]) could have accounted for total overprinting of significantly ^13^C- and ^18^O-depleted primary signals with marine ones in the analysed rugosans. Once stabilized to low-Mg calcite during early diagenesis, in turn, carbonates are little prone to further alteration in both calcite-saturated seawater and the rock-buffered, late diagenetic realm [61,63,73].

Furthermore, while oxygen is considered to be susceptible to re-mobilisation in near-surface diagenetic settings, very high water-rock ratios (i.e., open-system diagenesis) must be involved to shift considerably the carbon isotope composition of carbonate minerals [70,71,74,75]. This is difficult to accomplish not only in late diagenetic environments, but also in shallow sediment pore waters; interstitial waters in modern deep-seas are usually nearly stagnant [76], and this was presumably more pronounced in the Palaeozoic, when the activity of burrowing organisms was considerably less intensive [77]. Finally, preservation of the ghosts of the original skeletal structures (*M*. *praecox*, *M*. *roberti* from Madène el Mrakib) or even original growth increments (*C*. *sandalina*) is possible only in the case of solution-precipitation reactions taking place at a scale smaller than the size of the pristine skeletal elements, thus attesting for closed or semi-closed system recrystallisation and hence little exchange between skeletal carbonate and external diagenetic fluids [60,61,78–80]. A closed to semi-closed diagenetic system was facilitated by the shaly to marly character of the host sediments, as fine-grained deposits hamper fluid movement and often serve as effective barriers to diagenetic fluid circulation (e.g., [19,63,78]).

#### Case observations

**Fig 7:**
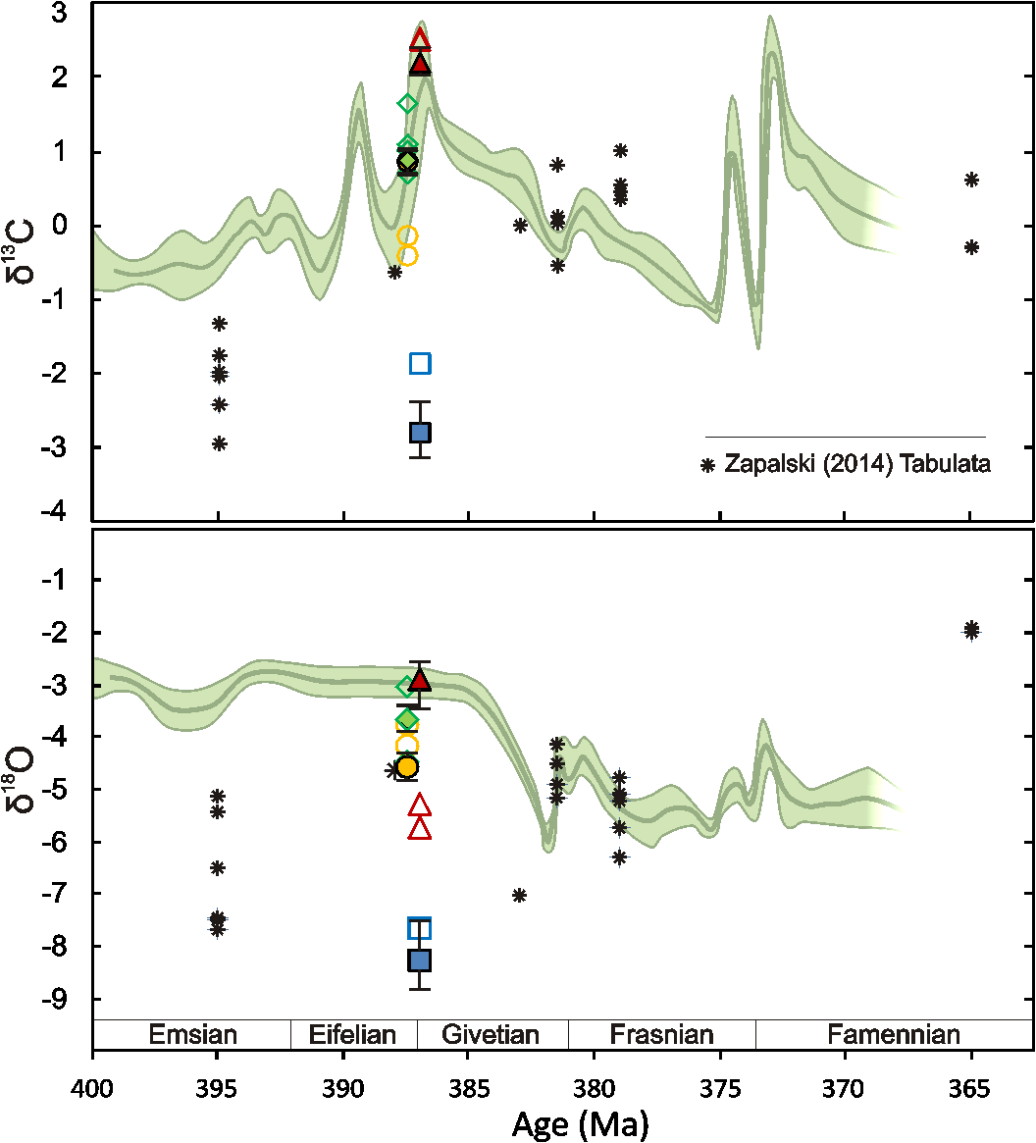
Comparison of the δ^13^C and δ^18^O values observed in the studied Devonian rugosans with Devonian tabulate coral data of Zapalski [17], and carbon (A) and oxygen (B) isotopic curves for Devonian marine calcites according to van Geldern et al. [31]. The curves were produced as Locfit trend lines with indicated 95% confidence intervals based on stable isotope analyses of non-luminescent brachiopod shells (see [31] for details). See Fig 5 for symbols; data of Zapalski [17] are indicated with asterisks. For the least-altered samples, each symbol depicts average value of the signatures measured, with vertical lines corresponding to the highest/lowest values. All results are given in ‰ V-PDB.

The isotope patterns observed in the studied corallites are in good agreement with the above outlined considerations. Except for the *M*. *tortuosus* specimen from Aferdou el Mrakib, all the rugosans show narrow clustering of the δ^13^C signatures, falling within the ranges of calcites precipitating in equilibrium with contemporaneous seawater, and this is the case also for the δ^18^O values found in *P*. *porpitus* from Gotland, *M*. *roberti* from Madène el Mrakib, and, most likely, also *C*. *sandalina* from the Holy Cross Mountains (Fig 7; see [81] for Silurian, and [31] for Devonian stable isotope values of marine carbonates inferred from brachiopods).

While the *P*. *porpitus* corallite was strongly affected by recrystallisation, the case of Madène el Mrakib specimen is particularly remarkable. This *M*. *roberti* corallite comes from exactly the same locality as brachiopods used by van Geldern and co-workers [31] to construct the Middle Devonian portion of their isotopic curves, so that, given the relatively narrow range of δ^18^O values found in the Madène el Mrakib brachiopods [31], it can be confidently identified as bearing a marine signal (Fig 7). The Madène el Mrakib specimen has still visible original structural elements and entirely lacks luminescence, which is usually indicative of relatively good preservation (e.g., [15,31,82]). Indeed, the low level of geochemical alteration and associated preservation of the primary stable isotope signals of this corallite is consistent with its low Mn and Fe contents, and with the highest concentrations of Sr and Mg among the investigated coral specimens; both Sr and Mg contents in carbonates tend to decrease with diagenetic alteration [58,82,83]. The non-luminescence of the *M*. *roberti* specimen makes alteration in the presence of early-burial, marine-derived pore fluids very unlikely. Meteoric diagenesis, which may result in obliterating the primary isotopic signatures with preservation of the non-luminescence, can be, in turn, excluded given the depositional history of the Mader Basin [42], as well as the lack of ^18^O-depletion of the corallite except for its outermost, luminescent portions. In these areas, the low δ^18^O values can be most likely attributed to increased temperature of recrystallisation, which is the most important factor influencing oxygen isotope ratios of carbonates in the marine burial realm [84]. Overall, both the δ^13^C and δ^18^O values found in the least-altered portions of the Madène el Mrakib corallite are interpreted here as the primary signals acquired by the coral during its growth.

The *C*. *sandalina* and *M*. *praecox* specimens from the Holy Cross Mountains show δ^13^C values matching closely to the uppermost Eifelian marine signatures reported by van Geldern et al. [31], but their δ^18^O signals fall below the van Geldern et al.'s [31] brachiopod-based oxygen isotopic curve (Fig 7). For *C*. *sandalina*, it seems that its slightly ^18^O-depleted signatures as compared to the signals of *M*. *roberti* from Madène el Mrakib can be attributed to different temperature and/or oxygen isotope composition of seawater from which the corallites were precipitated, rather than to any secondary diagenetic effect. While the Mader Basin was situated at relatively high latitude during the Middle Devonian (ca. 35–40°S, according to [32]; Fig 1), the Holy Cross Mountains were located at that time much closer to the equator, and the δ^18^O values measured in the *C*. *sandalina* specimen indeed fall well within the range of signatures reported for Middle Devonian low-latitude brachiopods from elsewhere ([15,31,72,85]; see also [42]). This is in agreement with the textural observations; while there is still some disagreement as to the origin of the lamellar structures found in Palaeozoic corals (e.g., [24,25,86,87]), it seems beyond doubt that these features represent at most minor level of early-diagenetic recrystallisation, resulting in the fusion of neighbouring fibrous crystals, but preserving the original growth increments [23,24,87]. Some degree of diagenetic restructuring appears suggested by the Fe-enrichment and dull luminescence of the well-laminated parts of the *C*. *sandalina* skeleton. Nevertheless, the luminescence and somewhat increased Fe concentrations as such are not decisive arguments against the primary nature of lamellar structures, as both can be present in relatively unaltered biotic and abiotic marine carbonates (e.g., [56,57,88]). The presence of luminescence was also observed in apparently very well-preserved scleractinian corals [89]. In any case, such minor recrystallisation, with transfer of material only within, but not between different laminae, is of limited potential in considerable shifting the primary stable isotope values.

Some depletion in ^18^O observed in the *M*. *praecox* corallite can be most probably attributed to its alteration in the presence of somewhat increased temperature. This is in agreement with the luminescence pattern of the *M*. *praecox* skeleton, as well as with its considerable enrichment in both Mn and Fe, combined with its depletion in Mg and Sr. In such a scenario, the outer, brightly luminescent portions of the corallite could have stabilized first during interactions with early diagenetic, relatively cold fluids enriched in ^12^C due to decomposition of organic matter, whereas the dull-luminescent parts would have stabilized slowly in the presence of progressively warmer diagenetic solutions. The increased temperature of these later diagenetic fluids is in accordance with the low δ^18^O values measured also in the outermost parts of the *C*. *sandalina* corallite. Since changing of carbon along with the oxygen isotope composition would require very high/water rock ratios, unexpected given the presence of very well-defined remnants of the original structures, the negative correlation between δ^13^C and δ^18^O found for the *M*. *praecox* specimen can be, in turn, explained most likely by addition of small amounts of early-diagenetic cements, rather than directly to diagenetic recrystallisation. Even corals entirely devoid of macroscopically-visible intraskeletal spaces, such as those analysed in the present approach, still have numerous micropores in their skeletons left by decomposition of organic matrix (cf., [29,90]). Cementation of these minute open spaces can result in small, yet noticeable shifts in stable isotope composition of corallites [12,28], similar to those observed in the *M*. *praecox* specimen. Regardless, a primary origin of this co-variance seems implausible, as in such a case vital fractionation mechanisms exhibited by rugosans would have to be very different than those known not only from modern scleractinians, but also from other marine invertebrates, in which metabolic and/or kinetic fractionation processes always result in a positive correlation between δ^13^C and δ^18^O [7,8,57,91].

Based on the microscopic and elemental analyses, the corallites of *P*. *porpitus* from Gotland and *M*. *tortuosus* from Aferdou el Mrakib can be identified as the most altered specimens analysed in the present study. However, the isotope ratios of *P*. *porpitus* are in the range of local Telychian brachiopod calcite values [81,92]. Most probably, this can be attributed to recrystallisation during early burial in the presence of shallow interstitial fluids that had carbon and oxygen isotope ratios similar to those of seawater. The strongly negative δ^13^C and δ^18^O values displayed by the *M*. *tortuosus* specimen, significantly offset from the composition of contemporaneous marine calcite [31], are, in turn, interpreted here as diagenetic signatures. Strong depletion in ^18^O and less pronounced depletion in ^13^C are the patterns commonly observed in carbonates recrystallised in both meteoric and marine diagenetic settings, but differ remarkably from the δ^13^C and δ^18^O signatures measured in modern ahermatypic, azooxanthellate Scleractinia, in which, besides the co-variance of carbon and oxygen isotope ratios, δ^13^C values are significantly more affected than δ^18^O signals [10,55,59]. Somewhat surprisingly, the Aferdou el Mrakib specimen shows low Mn and Fe contents and a dark blue luminescence response, most often observed in unaltered carbonates [73,80,88,93]. Given the sedimentary environment and depositional history of the Mader Basin during the Devonian [41], the possibility of meteoric diagenesis as responsible for this luminescence pattern can be excluded. Perhaps, the low Mn and Fe concentrations can be interpreted here as resulting from a lack of sufficient quantities of both Mn^2+^ and Fe^2+^ in the diagenetic fluids present at a time of the corallite recrystallisation (cf., [82,83,94]). Accordingly, the Aferdou el Mrakib corallite shows both a significantly higher level of textural restructuring, as well as considerably lower Mg and Sr contents than the other of the analysed non-luminescent corals, i.e., the Madène el Mrakib specimen, thus attesting more advanced diagenetic alteration. The blue luminescence alone, in turn, is not indicative of the pristine preservation, since carbonates usually luminesce blue due to the presence of lattice defects, with no direct relationship to either Mn or Fe contents [93–95]. These observations are in line with other studies implying that the use of luminescence patterns as the main preservation test must be implemented with caution (e.g., [15,75,82,94]).

### Implications for stable isotope studies of Palaeozoic corals

Since most previous analyses of stable isotope compositions of modern and fossil cnidarians were performed on aragonitic skeletons of Scleractinia, past investigations of the isotopic geochemistry of Palaeozoic corals were influenced mostly by scleractinian studies. As a result, low isotope ratios found in some rugosan skeletons were interpreted as primary signals resulting from fractionation processes analogous to those observed in modern scleractinians [13], and the lack of correlation between δ^13^C and δ^18^O values was even suggested to provide evidence for the presence of photosymbionts in tabulate coral tissues [17]. Likewise, the lack of ^13^C depletion, and only slight—if any—depletion in ^18^O observed in the Famennian rugose coral *Scruttonia kunthi* was found by Berkowski and Belka [16] puzzling and interpreted with caution.

Direct comparison between the results of the present and previous studies of isotopic geochemistry of Palaeozoic corals is somewhat difficult, since most of the former analyses either concentrated on various carbonate components of marine sediments with no particular focus on corals [13–15], or concerned a different group of corals (i.e., Tabulata) [17]. Berkowski and Belka [16] studied a rugose coral, but that species was, in turn, colonial and inhabited a shallow-water environment, and thus, by analogy with modern scleractinians, could have been typified by different isotopic fractionation effects than deep-water forms (cf., [6,10,58]).

The results of the present study are in agreement with previous investigations of rugose corals by Popp et al. [15] and Berkowski and Belka [16], who observed no significant vital fractionation effects in rugosan skeletons. It seems also in line with the data of Brand and Veizer [14], who found the rugosan stable isotope signals to be similar to those of contemporaneous brachiopods (yet, under the influence of the previous work of Brand [13], this was controversially attributed to diagenetic alteration therein). In particular, in the work of Berkowski and Belka [16] carbon isotope signals were found to be very uniform throughout many years of skeleton growth, whereas δ^18^O signatures did show noticeable variability related probably to seasonal environmental changes, but still within the range of values reported for calcites precipitated in equilibrium with Famennian seawater.

The only study that showed pronounced depletion in ^18^O (but, remarkably, at most slight depletion in ^13^C) in rugosans was that of Brand [13], who investigated the deep-water species *Lophophyllidium profundum* from the Pennsylvanian Kendrick shale (Kentucky). The origin of this ^18^O-depletion—contrasting with the roughly marine values found in rugosans by Brand and Veizer [14], Popp et al. [15], Berkowski and Belka [16] and in the present study—remains unclear. If one assumes the pattern reported by Brand [13], with the lack of positive δ^13^C-δ^18^O correlation and only minor, if any, ^13^C-depletion, to represent the primary signatures, then the vital effects imposed by rugosans would have to be very unlike those known from modern azooxanthellate scleractinians. Perhaps, as in the case of the Aferdou el Mrakib specimen analysed herein, the ^18^O-depletion can be, therefore, attributed to diagenetic alteration. This seems at odds with the apparently very good preservation state advocated by Brand [13] for the fossils from the Kendrick shale, and, in particular, good preservation of aragonitic skeletons in the studied succession. It must be noticed, however, that it is microstructure, porosity, organic matter content and surface-to-volume ratio, and not only skeletal mineralogy alone that constitute decisive factors in the pattern of alteration of carbonate materials [69,78]. Hence, the preservation of some fossils as aragonite does not automatically preclude isotopic exchange between porous, organic- and Mg-enriched corallites and diagenetic solutions in the case of oxygen isotopes, which can be mobilized prior to any other commonly measured geochemical parameters [70,84]. Besides, as noticed by Brand [27], the presence of diagenetic cements in the intraskeletal spaces and on the external walls of the Kendrick corals (see also [19]) could have resulted in shifting the isotopic values in the studied corals.

The absence of isotopically depleted stable isotopic signatures and the lack of correlation between δ^13^C and δ^18^O in ahermatypic, non-photosymbiotic rugosans shown in the present study have important implications also for studies of Palaeozoic tabulate corals, such as the recent work of Zapalski [17]. While tabulate corals differed from rugosans in several morphological and ecological aspects, they generally revealed similar mineralogy and microstructural features (e.g., lamellar structures) [23–25] that are not observed in modern scleractinians. Although detailed case studies on tabulates are obviously needed, our results show that the lack of the δ^13^C-δ^18^O co-variance in Palaeozoic corals cannot be interpreted as indicative of their photosymbiotic lifestyle, as argued by Zapalski [17]. It is also evident that one cannot interpret the isotopic signatures of Palaeozoic corals by presenting them on a diagram designed for Scleractinia, such as that of Stanley and Swart [1], used by Zapalski [17] to argue after the presence of photosymbionts in Tabulata. The present study underlines the importance of discussing measured stable isotope values in comparison with signals of contemporaneous marine carbonates, which, in the case of the Devonian, were significantly more depleted in ^18^O than they are today. Had such a correction been done by Zapalski [17], many of his data would fall within or close to (within ±1‰) the isotopic signatures of Devonian marine calcite (Fig 7), and thus above the 'warm non-zooxanthellate line' of Stanley and Swart [1], providing no indication of tabulate-algae cooperation. The only Devonian tabulates investigated by Zapalski [17] that display real depletion in both ^13^C and ^18^O (Fig 7) are, in turn, Emsian *Favosites* colonies from the Holy Cross Mountains (Poland) and the Hamar Laghdad area (Morocco); remarkably, in the latter case the corals inhabited relatively deep-water, hemipelagic settings inferred to be aphotic (e.g., [96,97]). Given the susceptibility of delicate tabulate corallites to diagenetic restructuring, these low δ^13^C and δ^18^O values could have originated due to diagenetic alteration or sampling of intraskeletal cements along with the thin-walled and porous skeletons of tabulates.

In the absence of demonstrably primary depletion in ^18^O, the high δ^13^C signals found in Palaeozoic corals cannot serve as indication of the coral-algae symbioses, as they are used in studies of modern scleractinians. Palaeoecological arguments [17,22] notwithstanding, the question of the possible presence of photosymbionts in tissues of both Rugosa and Tabulata cannot be, therefore, elucidated based on the available stable isotopic data. Despite some previous suggestions [17], the invariable, marine carbon isotope signatures found in massive, layered, shallow-water colonies of the rugosans analysed by Berkowski and Belka [16] are unlike those typical of modern massive, banded zooxanthellate scleractinians, characterized by some (though less pronounced than in azooxanthellate corals) ^13^C-depletion and variable δ^13^C values of individual growth bands, reflecting temporal fluctuations in the activity of photosymbionts (e.g., [4,10,59]).

The results of the present approach add to a growing number of characteristics known to distinguish Palaeozoic Rugosa from modern Scleractinia, such as skeletal mineralogy, septal symmetry, and possibly also some microstructural features [23,98–101]. In general, this should not be found surprising. Studies by Romano and Palumbi [102], Ezaki [103], Stolarski et al. [104] and Park et al. [105] provided evidences that Scleractinia evolved from soft-bodied ancestors that diverged from other Cnidaria probably not later than in the Early Palaeozoic, and thus must have developed their skeleton secretion mechanisms independently of other calcareous cnidarians. Palaeozoic corals cannot be, therefore, arbitrarily assumed as more similar in their skeletal isotopic composition to Scleractinia than to other groups of extant skeleton-forming cnidarians, i.e., Octocorallia and Hydrocorallia. Remarkably, modern, Mg-calcitic octocorals, as well as aragonitic hydrocorals display either no appreciable non-equilibrium isotopic fractionation effects [6,106–108], or conspicuously less pronounced isotopic depletion [109,110]. This shows that the very strong kinetic fractionation effects peculiar to scleractinians and distinguishing them from other groups of marine invertebrates [7,8,54], can by no means be considered typical of all calcifying Cnidaria. In terms of carbon and oxygen isotope fractionation, Palaeozoic rugose corals seem, therefore, similar to most modern marine biota (including non-scleractinian corals), with respiratory effects being more important than kinetic effects.

## Conclusions

The results of our study show that, having specimens with still distinguishable primary microstructural features, suitable luminescence and trace element characteristics, and embedded in sediments hampering diagenetic fluid movement and fluid-rock interactions, it is possible to obtain primary stable isotope signals from corallites of rugose corals. Based on these signatures, four main conclusions can be drawn:

- The coral specimens identified as best preserved on the basis of multi-proxy preservation tests show marine or near-marine δ^13^C and δ^18^O values. While we cannot completely exclude that rugose corals did impose minor vital fractionation effects, it is clear that isotopic fractionation effects exhibited by rugosans must have been considerably different—and much less pronounced—than those of modern aragonitic, scleractinian corals. Rather, the isotopic composition of calcitic rugosans seems similar to other marine invertebrates, including two other groups of extant calcifying cnidarians, i.e., Mg-calcitic octocorals and aragonitic hydrocorals.
- Pronounced differences between stable isotope signatures of the inner, better-preserved, non- or dull-luminescent portions of the skeletons, and outer, strongly recrystallised, orange-luminescent parts of the corallites indicate that investigative techniques, such as transmitted-light, cathodoluminescence, scanning electron microscope, and/or trace element analyses need to be implemented in order to assess the preservation state of studied coral materials, before any interpretations of their potential primary isotopic signatures can be made.
- Non-photosymbiotic rugose corals do not show correlation between δ^13^C and δ^18^O, and thus the lack of such cannot be used as evidence of photosymbiosis in Palaeozoic rugosans. By inference, this applies also to tabulate corals (cf., [17]), unless the presence of δ^13^C-δ^18^O co-variance is reliably demonstrated. Consequently, as without such a correlation in non-photosymbiotic corals neither ^13^C-enrichment nor ^18^O-depletion is indicative of the presence of photosymbionts in shallow-water corals, distinguishing between symbiotic vs. non-symbiotic mode of feeding in Palaeozoic cnidarians based on their stable isotope composition may not be possible.
- Despite the indications that rugosan corallites can bear marine isotopic signals, the wider use of rugose corals as proxies of former seawater composition is not recommended here. The main problems are the general susceptibility of rugosan skeletons to at least some level of diagenetic alteration, associated with their intermediate-Mg-calcitic mineralogy, the presence of intraskeletal organic matrices, as well as the presence in most species of large intraskeletal open spaces filled with cements, often difficult to distinguish from the actual skeletal materials. In each case, the results must be interpreted with caution.

## Supporting Information

S1 Fig Middle Palaeozoic stratigraphic chart with indicated stratigraphic positions of the studied specimens. (PDF)
