# Correction: Stable Isotope Signatures of Middle Palaeozoic Ahermatypic Rugose Corals—Deciphering Secondary Alteration, Vital Fractionation Effects, and Palaeoecological Implications

**DOI:** 10.1371/journal.pone.0140199

**Published:** 2015-10-09

**Authors:** PLOS ONE Staff

There are errors in the “Locality” column of Table 1. The publisher apologizes for the errors. Please see the corrected Table 1 here.

**Table 1:** Taxonomy, localities and the most important textural features of the studied coral specimens.

Genus	Species	Spec. #	Age	Locality	Textural preservation	Luminescence pattern	Figures
*Microcyclus*	*praecox*	UAM Tc/B/SK/20	uppermost Eifelian (*ensensis* Zone?)	Skały, Holy Cross Mountains, Poland (50°53'43.76'' N, 21°09'32.53'' E)	Lower part with numerous ghosts of original structures (septae with centres of calcification, interseptal stereome); upper part strongly recrystallized	Lower part dully (red) luminescent, upper part brightly luminescent	2A-C, 3A-F, 6A-B
*Microcyclus*	*roberti*	UAM Tc/B/MM/01	lowermost Givetian (*hemiansatus* Zone)	Madène el Mrakib, Mader Basin, Morocco (30°44'03.11'' N, 4°42'15.52'' W)	Central part with recognizable former centres of calcification and interseptal stereome; outermost portions strongly recrystallized	Non-luminescent except for the outermost part (brightly luminescent)	2D-F, 3G-L, 6D-F
*Microcyclus*	*tortuosus*	UAM Tc/B/AM/01	lowermost Givetian (*hemiansatus* Zone)	Aferdou el Mrakib, Mader Basin, Morocco (30°45'50.76'' N, 4°37'12.87'' W)	Recrystallized with few traces of original textural elements	Dark-blue luminescent except for the outermost part and borings (brightly luminescent)	2G-I, 4A-C, 6G-I
*Palaeocyclus*	*porpitus*	UAM Tc/B/GO/01	Telychian	Visby, Gotland, Sweden (57°35'24'' N 18°11'01'' E)	Strongly recrystallized with a lack of recognizable original structures	Brightly (red to orange) luminescent	2J-L, 4D-F, 6M-N
*Calceola*	*sandalina*	UAM Tc/B/SK/50	lowermost Givetian (*hemiansatus* Zone)	Skały, Holy Cross Mointains, Poland (50°53'43.76'' N, 21°09'32.53'' E)	Inner parts with well-defined growth increments and laminar ultrastructure; outer parts recrystallized	Inner part dully luminescent, outer part red luminescent	2M-O, 4G-L, 6J-L
