# Supplementary material for: Stable Isotope Signatures of Middle Palaeozoic Ahermatypic Rugose Corals – Deciphering Secondary Alteration, Vital Fractionation Effects, and Palaeoecological Implications
Source: PLoS One. 2015 Sep 3;10(9):e0136289. doi: 10.1371/journal.pone.0136289 (PMC4559396; doi:10.1371/journal.pone.0136289)
Supplement: S1 Fig — (PDF) [file pone.0136289.s001.pdf]

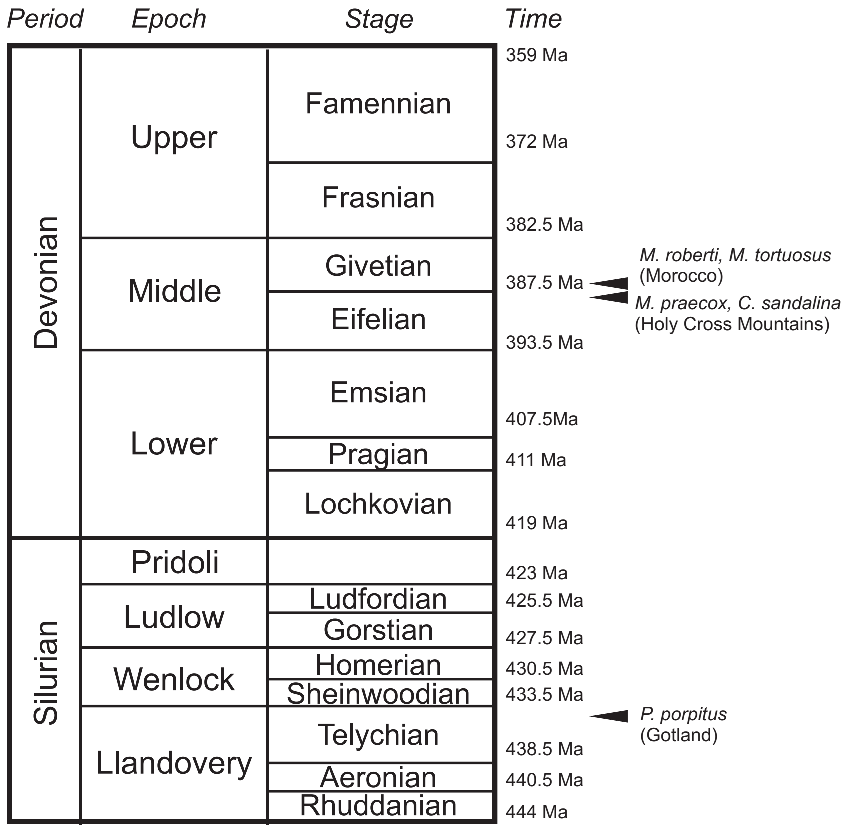

S1 Figure. Middle Palaeozoic stratigraphic chart (after Cohen et al., 2013), with indicated stratigraphic positions of the studied specimens.

Reference

Cohen KM, Finney SC, Gibbard PL, Fan J-X. The ICS International Chronostratigraphic Chart. Episodes. 2013 (updated); 36:199-204.
